# A survey of steroid-related osteoporosis diagnosis, prevention and treatment practices of pediatric rheumatologists in North America

**DOI:** 10.1186/1546-0096-12-24

**Published:** 2014-07-09

**Authors:** Arzu Soybilgic, Melissa Tesher, Linda Wagner-Weiner, Karen B Onel

**Affiliations:** 1 University of Illinois at Chicago, Chicago, IL, USA

**Keywords:** Pediatric, Rheumatology, Systemic, Juvenile, Idiopathic, Arthritis, Osteoporosis, Corticosteroids, North America

## Abstract

**Background:**

The purpose of our study is to assess practices of North American pediatric rheumatologists regarding monitoring, prevention, and treatment of low bone mineral density (BMD) in children on long-term glucocorticoid treatment.

Long-term glucocorticoid therapy is associated with accelerated bone loss. Children with JIA and lupus have low baseline BMD and incident vertebral fractures commonly occur in these groups of patients even after a relatively short period of time being on systemic glucocorticoids. There are no established guidelines for identification, prevention, and treatment of glucocorticoid-induced bone loss in children.

**Methods:**

A cross-sectional online survey was conducted with 199 physicians who were listed in the ACR database as practicing pediatric rheumatology in North America.

**Results:**

86 physicians (43%) responded; 87% were board-certified in pediatric rheumatology. 95% used dual energy X-ray absorptiometry as their primary modality for assessing BMD. 79% “rarely” or “never” obtained a baseline BMD measurement prior to initiation of glucocorticoid therapy. 42% of respondents followed BMD annually. 93% “frequently” or “always” prescribed calcium for patients on long-term corticosteroid therapy; 81% “frequently” or “always” prescribed vitamin D. In patients diagnosed with osteoporosis, 35%-50 % of the practitioners “sometimes”, “frequently” or “always” prescribed bisphosphonates. Bisphosphonates are prescribed at similar rates for male and female patients, and slightly more frequently for pubertal than for pre-pubertal patients. 96% of respondents “rarely” or “never” prescribed calcitonin for patients on long-term glucocorticoid therapy; 92% “rarely” or “never” prescribe this medication for patients with known osteopenia or osteoporosis.

**Conclusions:**

Utilization of DXA in children on long-term corticosteroid therapy varies greatly among North American pediatric rheumatologists. Most respondents do not screen for low BMD on a regular basis despite acknowledging the risks of bone loss in this population. Broad consensus appears to be present among practitioners favoring the prescription of calcium and vitamin D for patients receiving long-term corticosteroid therapy. Relatively few respondents consistently recommend bisphosphonate therapy, even for patients with known low bone density; calcitonin is rarely used. These data underscore the need for studies to acquire specific data on bone loss, and its prevention and treatment in young patients on long-term glucocorticoid therapy.

## Background

Long-term glucocorticoid therapy is known to be associated with accelerated bone loss, causing increased risk of osteoporosis and fracture [1]. Low bone density is common in juvenile idiopathic arthritis (JIA), lupus and other pediatric rheumatologic diseases [2-7]. Many patients with JIA have low BMD, even at baseline before the start of systemic glucocorticoid treatment. 30% of mildly to moderately ill pre-pubertal JIA patients who had never been treated with corticosteroids had low total body BMD [8]. Asymptomatic vertebral fractures are an under-recognized problem in children with inflammatory disorders and can be present prior to prolonged glucocorticoid exposure in pediatric rheumatology patients [9,10].

The ACR has published clinical guidelines for diagnosis and management of osteoporosis for adults on long-term glucocorticoid therapy [11]. Despite guidelines, some studies show that many patients treated with glucocorticoids do not receive any treatment to prevent bone loss [12,13]. A recent survey of adult rheumatologists revealed significant variability in corticosteroid – induced osteoporosis management [14]. There is even less experience on this medical issue in pediatrics, as there are no established clinical guidelines for identification and prevention of glucocorticoid-induced bone loss in children. Furthermore, once the diagnosis of osteopenia/osteoporosis is established in children, treatment options are limited and poorly studied.

There are several studies reporting that bisphosphonates are safe and effective in children [15-21]. However, there are still concerns among pediatric rheumatologists about the long-term effects of bisphosphonate use in children. Pediatric rheumatologists hesitate to prescribe bisphosphonates to children because the possible long-term adverse effects, the necessary length of treatment and the maximal bone mass gain that can be achieved in children are all unknown.

With this survey, we aimed to assess current practices of North American pediatric rheumatologists regarding monitoring, prevention, and treatment of osteoporosis and osteopenia in children on long-term glucocorticoid treatment.

## Methods

### Sample population

Using electronic mail, an online, self-administered survey was sent to 199 physicians practicing pediatric rheumatology in North America. Physicians were surveyed using a questionnaire probing the diagnosis, prevention, treatment, and monitoring of osteoporosis in children receiving long-term oral systemic corticosteroid therapy.

Ethics approval was granted by the University of Chicago Institutional Review Board.

### Survey design

The survey was comprised of 17 items (see Additional file 1). It addressed three major themes in management of glucocorticoid-induced osteoporosis in children: 1) Preferred methods of diagnosis 2) Current approaches to prevention 3) Preferred treatment choices.

### Analysis

Data analysis included descriptive statistics applied to the closed-ended questions, expressed as frequency (percentages) for categorical variables.

## Results

86 of the 199 physicians who were sent the survey completed the survey (43% response rate), of which 85% were board certified to practice pediatric rheumatology in the US. 84% of the respondents were pediatric rheumatologists, 10% were medicine/pediatric rheumatologists, 1% pediatric rheumatology fellow, 1% adult rheumatologist, and 4% listed themselves as “other”. The most common practice setting, with 86%, was an “Academic Medical Center”. 5% of the respondents practiced at a community hospital, 5% in private practice, and 4% reported their practice setting as “other”.

46% of the physicians reported that they would consider measures to identify or prevent corticosteroid-induced bone loss in patients treated with any steroid dose. 31% would consider such measures only at prednisone doses greater than 0.5 mg/kg/day, 17% at doses greater than 1 mg/kg/day, 5% at doses greater than 2 mg/kg/day.

80% of the physicians never or rarely obtained a baseline BMD measurement, by any method, before initiating long-term (>6 months) glucocorticoid treatment; 11% did so sometimes, 7% frequently. Only 2% of the physicians always obtained a baseline measurement before initiating long-term glucocorticoid treatment.

The most commonly used tool for initial BMD measurement and to monitor steroid-related bone loss was DXA (96%); 4% of the physicians used quantitative CT, and none of the responders used quantitative ultrasound of bone or single X-ray absorptiometry of bone for these purposes. 5% of the physicians obtained follow-up BMD measurements every 6 months, 42% annually, 5% every 18 months, and 16% less frequently than every two years. 2% never obtained follow-up BMD measurements. Only 6% of physicians reported having a written policy in their department regarding the frequency of BMD measurements in children on long-term glucocorticoid treatment.

93% of the physicians reported starting calcium supplements frequently or always for patients who are on long- term corticosteroid treatment. Vitamin D supplements were given frequently or always by 80% of physicians. 40% of the responders prescribed combined calcium/vitamin tablets.

In patients diagnosed with either osteopenia or osteoporosis, between 35% and 50% of the practitioners “sometimes”, “frequently” or “always” prescribed bisphosphonates. Bisphosphonates are prescribed at similar rates for male and female patients, and slightly more frequently for pubertal than for pre-pubertal patients. 96% of respondents “rarely” or “never” prescribe calcitonin for patients on long-term glucocorticoid therapy, and 92% “rarely” or “never” prescribe this medication for patients with known osteopenia or osteoporosis.

## Discussion

Low bone density is common in children with JIA, lupus and other rheumatologic diseases [2-5]. 30% of mildly to moderately ill pre-pubertal JIA patients who had never been treated with corticosteroids had low total body BMD [8]. Patients with juvenile SLE have a low bone mass without catch-up growth over time, causing a reduction of peak bone mass with high risk of osteoporosis in early adulthood [7]. Local inflammatory factors, decreased physical activity, nutritional imbalances, and medications have all been implicated in bone loss [22,23]. In our study, many physicians (46%) reported that they would consider measures to identify or prevent corticosteroid-induced bone loss at any steroid dose. However, the preponderance (80%) of the physicians never or rarely obtained a baseline BMD measurement, by any method, before initiating long-term (>6 months) glucocorticoid treatment.

Vertebral fractures are an under-recognized problem in children with inflammatory disorders and they can be present prior to prolonged glucocorticoid exposure. A recent study reported that the incidence of vertebral fractures in children with rheumatologic diseases in the 12 months following glucocorticoid initiation was 6%, and most children were asymptomatic [10]. Another study on children with rheumatic conditions reported that 7% of children had vertebral fractures within 30 days of initiating glucocorticoids [9]. Studies in adults have shown that patients with glucocorticoid-induced osteoporosis experience fragility fractures at better DXA scores than those with postmenopausal or age-related osteoporosis. This might be explained, at least in part, by the effects of glucocorticoids not only on osteoclasts but also on osteoblasts and osteocytes [24]. Therefore, prevention and early diagnosis of low bone density is important in pediatric rheumatology patients to prevent fragility fractures.

Per the guidelines published by The International Society for Clinical Densitometry in 2013, in the absence of vertebral compression (crush) fractures, the diagnosis of osteoporosis in children is indicated by the presence of both a clinically significant fracture history and BMD Z-score ≤ -2.0. The Society stresses that diagnosis of osteoporosis in children and adolescents should not be made on the basis of densitometric criteria alone. However, they recommend that in patients with primary bone disease, or at risk for a secondary bone disease, a DXA should be performed when the patient may benefit from interventions to decrease their elevated risk of a clinically significant fracture, and the DXA results will influence that management. Per these guidelines, DXA is the preferred method for assessing BMC and areal BMD in children. Despite the data showing the high frequency of asymptomatic fragility fractures in children with rheumatologic diseases who are on long-term glucocorticoids and recommendations by The International Society for Clinical Densitometry summarized above, we found in our study that only 42% of the physicians obtained follow-up BMD measurements annually to screen for low bone density in patients who are on long-term glucocorticoid treatment. Few physicians (only 6%) had a written policy in their department regarding the frequency of BMD measurements in children on long-term glucocorticoid treatment.

In our study, DXA was the most commonly utilized diagnostic tool for diagnosis and follow-up of glucocorticoid-induced osteoporosis. None of the physicians who responded to our survey reported using bone ultrasound to monitor bone health in pediatric rheumatology patients who were on long-term glucocorticoid treatment. However, there is a growing body of evidence regarding the use of bone ultrasound in children to monitor bone density. Quantitative ultrasound (QUS) can be used for radiation-free assessment of bone density and “bone quality” by measurement of the ultrasound wave attenuation by bone (BUA) in children with JIA. Bone speed of sound (SOS) shows a significant correlation with BMD as measured by DXA. Portability, ease of use, lower cost, and absence of radiation make QUS of bone a promising means of evaluating BMD in children [25-31].

As stated earlier, there are no guidelines for prevention or treatment of glucocorticoid-induced osteoporosis in children. In our study, 93% of the physicians reported starting calcium supplements frequently or always as a preventative measure for patients who are on long- term corticosteroid treatment. Vitamin D supplements were given always or frequently by 80% of the respondents. In our study, 62% and 49% of the physicians never or rarely prescribed bisphosphonates for their pre-pubertal and pubertal female patients, respectively, with documented osteoporosis; 60% and 50% of the physicians never or rarely prescribed bisphosphonates for their pre-pubertal and pubertal male patients. A possible reason for its limited use in pediatrics is the concern of long-term side effects related to the persistence of bisphosphonates in bone. However, only bone tissue appears to be affected by bisphosphonates in vivo: the very rapid uptake by bone ensures that the other tissues are exposed for only a short period to these drugs (the half-life of circulating bisphosphonates in humans is 0.5-2 hours), and as far as we know, there is no evidence that bisphosphonates maintain any pharmacologic activity once sequestered in the bone [32]. In a prospective multicenter study, oral alendronate was found to be safe and efficacious for the treatment of osteoporosis in diffuse connective tissue diseases in children [15]. Several other studies to date have shown safety and efficacy of bisphosphonates in children [15,21,33,34]. Most commonly reported adverse effects included acute phase type of reactions and flu-like reactions (fever, muscle aches, bone pain) with IV infusion of bisphosphonates which usually responds to acetaminophen or ibuprofen and did not usually occur with further infusions [35-38]. Self-limited mild abdominal pain, nausea, and vomiting after the first infusion of bisphosphonates have also been reported [35]. Transient decreases in serum calcium and phosphate levels after treatment with intravenous pamidronate have been reported [16,17,39-41].

Several studies reported normal growth during treatment with bisphosphonates [15,36,37,42]. Metaphyseal banding (zebra lines) can be seen on the radiographs of children receiving cyclical bisphosphonate therapy [43]. The linear growth was found to be normal in several studies after use of bisphosphonates in children [17,44-46]. There were no effects of commonly used bisphosphonates on the growth plate and bone ages of children corresponded with their chronological age [17]. Bisphosphonate-associated osteonecrosis of the jaw has been reported in adults, but no cases have been reported in children so far [47].

There is a hesitance to use bisphosphonates in girls and young women of childbearing age due to concerns for potential unfavorable effects of bisphosphonate treatment on the fetus, mainly in the skeleton. Since bisphosphonates are retained for a long time in the human skeleton, concerns have been raised that even pre-pregnancy administration of bisphosphonates may result in embryo-fetal exposure and alter fetal bone modeling.

A recent study reviewed fifty-one cases of exposure to bisphosphonates before or during pregnancy; none of the pregnancies resulted in skeletal abnormalities or other congenital malformations in the infants [48].

Considering the high incidence of vertebral fractures in children on chronic glucocorticoid treatment and the data supporting safety and efficacy of bisphosphonates in children in general, it can be argued that the benefits of bisphosphonate therapy may outweigh the potential risks in patients with low bone density and frequent fragility fractures. Therefore, bisphosphonates should be considered as a treatment option for children who are on long-term glucocorticoid treatment and have documented osteopenia/osteoporosis.

Calcitonin is a naturally occurring peptide, which inhibits bone resorption by osteoclasts, and is useful for treatment of osteoporosis, reducing vertebral and hip fractures in adult patients. It is usually well tolerated, and its main side-effects are gastrointestinal. It can be administered by injection, orally or intranasally. Calcitonin has been shown to be safe and effective in treatment of steroid-induced osteoporosis in children with nephrotic syndrome [49]. Another recent study of intranasal calcitonin in 10 children with JIA showed a decrease in bone resorption markers and an increase in BMD [50]. Also, calcitonin can rapidly improve symptoms of vertebral fractures [51]. However, because the evidence is limited on the use of calcitonin in children, pediatric rheumatologists infrequently utilize it. 96% of respondents reported “rarely” or “never” prescribing calcitonin for patients on long-term glucocorticoid therapy, and 92% “rarely” or “never” prescribe this medication even for patients with known osteopenia or osteoporosis. More studies are needed in this area.

## Conclusions

Broad consensus appears to be present among North American pediatric rheumatologists favoring the prescription of calcium and vitamin D for pediatric patients on long-term corticosteroid therapy.

Despite the data showing the high incidence of osteoporosis and incident vertebral fractures in children on long-term glucocorticoids, relatively few respondents consistently recommend bisphosphonate therapy, even for patients with known osteopenia or osteoporosis, and calcitonin is rarely used. Furthermore, there is high variability of utilization of DXA in children on long-term corticosteroid treatment. These data underscore the need for more research on bone loss, and its prevention and treatment in young patients on long-term glucocorticoid therapy.

## Abbreviations

BMD: Bone mineral density; BMC: Bone mineral content; DXA: Dual-energy X-ray absorptiometry; SLE: Systemic lupus erythematosus; JIA: Juvenile idiopathic arthritis; Bone US: Bone ultrasound; SOS: Speed of sound.

## Competing interests

The authors declare that they have no competing interests.

## Authors’ contributions

AS conceived the study, AS, MT, LWW, KBO participated in its design, AS have collected the data and drafted the manuscript, MT, LWW, KBO have been involved in revising it critically for important intellectual content; all of the authors have given final approval of the version to be published and agree to be accountable for all aspects of the work in ensuring that questions related to the accuracy and integrity of any part of the work are appropriately investigated and resolved. All authors read and approved the final manuscript.

## Supplementary Material

Additional file 1 Survey questions. Click here for file
