# Supplementary material for: A survey of steroid-related osteoporosis diagnosis, prevention and treatment practices of pediatric rheumatologists in North America
Source: Pediatr Rheumatol Online J. 2014 Jul 9;12:24. doi: 10.1186/1546-0096-12-24 (PMC4105759; doi:10.1186/1546-0096-12-24)
Supplement: Additional file 1 — Survey questions. [file 1546-0096-12-24-S1.doc]

**SURVEY QUESTIONS**

1. This survey is specifically intended to assess practices of pediatric rheumatologists in North America. Do you practice mainly in North America?
   1. Yes
   2. No
2. Which answer best describes your current position?
   1. Pediatric Rheumatologist
   2. Pediatric Rheumatology Fellow
   3. Med/Ped Rheumatologist
   4. Med/Peds rheumatology fellow
   5. Adult Rheumatologist
   6. Adult Rheumatology Fellow
   7. Other
3. Are you board-certified in pediatric rheumatology?
   1. Yes
   2. No
4. Which answer best describes your primary practice setting?
   1. Academic Medical Center
   2. Community Hospital
   3. Private Practice
   4. Other
5. What is the approximate threshold dose of daily corticosteroids at which you begin to consider measures to identify or prevent corticosteroid-induced bone loss?
   1. >0.5 mg/kg/day
   2. >1 mg/kg/day
   3. 2 mg/kg/day
   4. Any dose
6. Which type of BMD measurement tool do you order most frequently?
   1. DXA
   2. QCT
   3. Single X-ray absorbtiometry
   4. Quantitative US of bone

# How frequently do you obtain a baseline BMD measurement, by any method, BEFORE initiating long term (>6 months) glucocorticoid treatment?

- 1. Never
  2. Rarely
  3. Sometimes
  4. Frequently
  5. Always

# How frequently do you obtain follow-up BMD measurements on patients who are ON LONG-TERM STEROID TREATMENT (>6 mo)?

- 1. Every 6 months, or more frequently
  2. Annually
  3. Every 18 months
  4. Every 2 years
  5. Less frequently than every 2 years
  6. Never

# Do you have a written policy in your department regarding the frequency of BMD measurements in patients on long-term glucocorticoid treatment?

# YES

- 1. NO

# How frequently do you start calcium supplements for patients who are on long-term corticosteroid treatment?

1. If you ever use CALCIUM supplements, WHAT DOSE do you typically recommend?
   1. < 500 mg/day
   2. 500 mg/day
   3. >1000 mg/day
   4. Other

# How frequently do you start VITAMIN D supplements for patients who are ON LONG-TERM CORTICOSTEROID TREATMENT?

- 1. Never
  2. Rarely
  3. Sometimes
  4. Frequently
  5. Always

# How frequently do you prescribe bisphosphonates for patients in the following groups, who are on LONG-TERM GLUCOCORTICOID THERAPY?

- 1. Never
  2. Rarely
  3. Sometimes
  4. Frequently
  5. Always

PRE-PUBERTAL FEMALE:

- 1. Never
  2. Rarely
  3. Sometimes
  4. Frequently
  5. Always

PUBERTAL FEMALE

1. Never
2. Rarely
3. Sometimes
4. Frequently
5. Always

PRE-PUBERTAL MALE

1. Never
2. Rarely
3. Sometimes
4. Frequently
5. Always

PUBERTAL MALE

1. Never
2. Rarely
3. Sometimes
4. Frequently
5. Always

# How frequently do you prescribe bisphosphonates for patients in the following groups, with KNOWN OSTEOPENIA OR OSTEOPOROSIS?

PRE- PUBERTAL Female

- 1. Never
  2. Rarely
  3. Sometimes
  4. Frequently
  5. Always

PUBERTAL Female

- 1. Never
  2. Rarely
  3. Sometimes
  4. Frequently
  5. Always

PRE-pubertal male

1. Never
2. Rarely
3. Sometimes
4. Frequently
5. Always

Pubertal Male

1. Never
2. Rarely
3. Sometimes
4. Frequently
5. Always

1. How frequently do you prescribe CALCITONIN to pediatric patients who are on long term glucocorticoid treatment? **...ARE on LONG TERM GLUCOCORTICOID TREATMENT?**
   1. Never
   2. Rarely
   3. Sometimes
   4. Frequently
   5. Always

1. How frequently do you prescribe CALCITONIN to pediatric patients who have known osteopenia/osteoporosis?
   1. Never
   2. Rarely
   3. Sometimes
   4. Frequently
   5. Always
